# Exceptional response to oral metronomic chemotherapy in a rare case of sinonasal mucosal melanoma

**DOI:** 10.3332/ecancer.2021.1287

**Published:** 2021-09-14

**Authors:** Anukul Karn, Satvik Khaddar, Amit Agrawal, Akhil Kapoor

**Affiliations:** Department of Medical Oncology, Mahamana Pandit Madan Mohan Malviya Cancer Centre and Homi Bhabha Cancer Hospital, Tata Memorial Centre, Varanasi, Uttar Pradesh, India 221005

**Keywords:** sinonasal melanoma, oral metronomic therapy, nasal cavity

## Abstract

Sinonasal mucosal melanoma is a rare malignancy with limited treatment options and a poor prognosis. They are usually diagnosed at an advanced stage owing to their late and nonspecific clinical presentation. Here we present a case of relapsed refractory unresectable malignant melanoma involving the right nasal cavity managed with Oral Metronomic Chemotherapy (OMCT). A 75-year-old male patient was diagnosed with unresectable malignant melanoma involving the right nasal cavity and showed radiological progression after initial management with four cycles of Dacarbazine. He was then shifted to OMCT (Cyclophosphamide, Celecoxib and Tamoxifen), as immunotherapy could not be given due to financial constraints, on which he showed approximately 40% reduction in tumour size after 6 months. This result can have important clinical implications in resource constrained settings, where the use of immunotherapy is limited by great financial burden.

## Introduction

Sinonasal mucosal melanoma is a rare and aggressive malignancy accounting for only 0.5%–2% of all melanomas. Its poor prognosis can be explained due to the fact that the development of symptoms is slow, leading to a delayed diagnosis, and the risk of local recurrence (31%–85%) and distant metastasis (25%–50%) is high. Diagnosis can be made using pathological and immunohistochemical findings [1]. The main modality of treatment remains surgery, with radiotherapy, chemotherapy and immunotherapy helping in controlling local and metastatic disease [1, 2]. Immunotherapy is not feasible in most of the patients in a resource limited setting either due to unavailability or due to cost constraints. Alternative chemotherapy regimens have been tried; however, due to the rarity of the disease, the knowledge of the management is limited to case reports, and since the patient responded to Oral Metronomic Chemotherapy (OMCT), the following case merits reporting.

## Case report

A 75-year-old male patient presented to our institute with chief complains of discharge from the right nasal cavity, rhinorrhoea and facial pain for the past 1 month before initial consultation. Diagnostic Nasal Endoscopy done under local anaesthesia showed a blackish mass in the right nasal cavity extending posteriorly. Nasopharynx and the left nasal cavity were free from the disease. Punch biopsy from the lesion was taken which showed an infiltrative tumour mass destroying the submucosal glands and sprinkled with melanin pigments, with tumour emboli in vessels along with areas of haemorrhage and necrosis. Tumour cells were positive for Human Melanoma Black-45 (HMB-45) on immunohistochemistry, confirming the diagnosis of right nasal cavity malignant melanoma.

MRI of the paranasal sinuses and neck showed a T1 hyperintense soft tissue mass of 8.3 × 2.7 cm epicentred in the right nasal cavity. Posteriorly it was extending into nasopharynx through choanae, inferiorly it was reaching up to the floor of nasal cavity, medially the mass was abutting nasal septum, superiorly it involved inferior aspect of both anterior and posterior ethmoidal sinuses with destruction of right-sided inferior and middle turbinate, uncinate process, and laterally, there was widening of maxillary ostium with no obvious extension into right maxillary sinus. No intraorbital or intracranial extension was seen. Positron emission tomography (PET) CT did not show any evidence of metastases. Staging based on American Joint Committee on Cancer 8th edition is Stage IVb (T4b, N0, M0).

**Figure 1.**
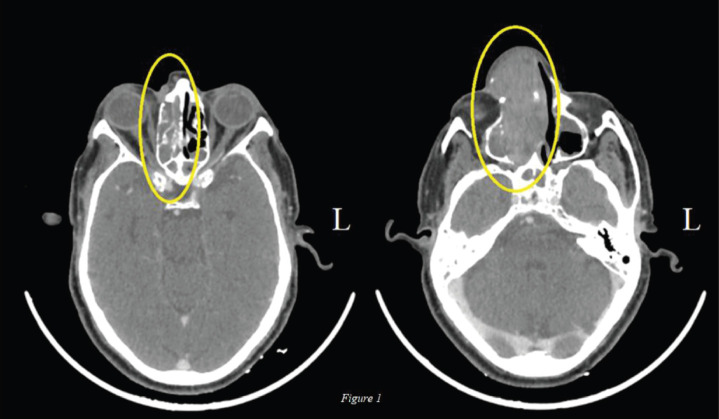
Axial view: Pre-OMCT CT of nasal cavity and sinuses: Interval increase in the mass (9.7 × 4.6 cm) involving right nasal cavity lesion creeping through nasopharynx to skull base without bony erosion, also inferomedially in right orbit and further in right paranasal sinuses.

**Figure 2.**
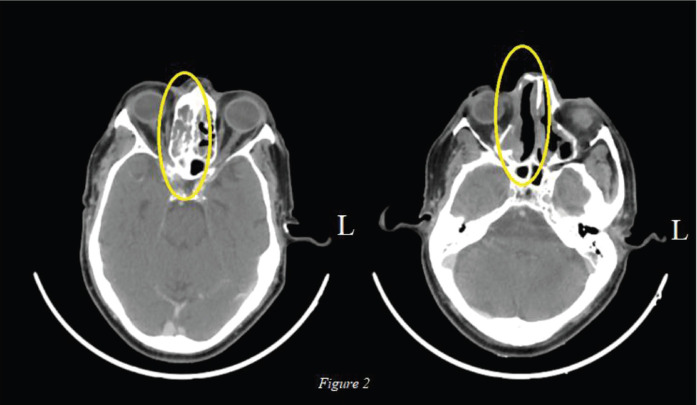
Axial view: Post 6 months of OMCT: Decrease in size and extent of the solid heterogeneously enhancing mass in the right nasal cavity to about 5.3 × 2.3 cm.

The lesion was deemed unresectable in view of superior extension of the intranasal mass and advanced loco-regional disease so the patient was started on palliative chemotherapy. The patient had an Eastern Cooperative Oncology Group (ECOG) Performance Score (PS) of 1 and was given four cycles of Dacarbazine. Post chemotherapy there was further increase in the size (9.7 × 4.6 cm), extent and effect of the right nasal cavity lesion creeping through nasopharynx to skull base without bony erosion, also inferomedially in right orbit and further in right paranasal sinuses (Figure 1). In view of primary progressive disease on first-line chemotherapy and preserved general condition, he was planned for second-line therapy; however, immunotherapy was not feasible due to financial constraints. Palliative radiation (30 Gy, two cycles per week for a total of four cycles with Intensity Modulated Radiotherapy (IMRT) technique using 6 MV Photons) was given for local control of the disease, however, the patient did not have any clinical benefit at 3 months of completion of radiotherapy and the scan showed stable disease after which the patient was planned for systemic chemotherapy. Intravenous chemotherapy was not feasible for the patient considering his age so he was planned for OMCT which consisted of Cyclophosphamide (50 mg once daily for 2 weeks followed by 1 week of drug free interval), Celecoxib (200 mg twice daily) and Tamoxifen (20 mg twice daily). At 3 months of follow-up, the patient had symptomatic benefit with reduction in pain and swelling over the nasal area. At 6 months, there was interval decrease in size and extent of the solid heterogeneously enhancing mass in the right nasal cavity to about 5.3 × 2.3 cm (42% decrease as per RECIST 1.1) suggestive of partial response (Figure 2). In view of symptomatic benefit and local control of the disease, the patient was continued on OMCT and currently is on a 3 monthly follow-up.

## Discussion

Malignant melanoma is a neoplastic transformation of melanocyte or melanocytic precursors commonly occurring on the skin, but can also occur in areas where neural crest cells migrate, like mucosal lining of gastrointestinal tract (nasal cavity of this patient) and brain [3]. Incidence of malignant melanoma is much higher in western (Europe (Age-adjusted rates in male: 26.7 and female: 31.7) and Americas (male: 13.4 and female: 10.9)) population, especially whites, as compared to Indians (male: 1.62 and female: 1.21) [3, 4].

According to literature review done by Alves *et al* [1] primary mucosal sinonasal melanoma corresponds to 0.5%–2% of all melanoma and approximately 4% of melanomas of head and neck. The mucosal melanoma is commonly seen in individuals ≥ 60 years (our patient was 75), commonly affected ‘the nasal cavity, septum, inferior and middle nasal conchae, the lateral wall of the nasal cavity and the facial sinuses’. More than 50% of patients have advanced disease at presentation, as also seen in our patient.

Sinonasal melanoma usually has a poor prognosis, as they are diagnosed at a much-advanced stage due to slow development of symptoms and due to increased risk of local recurrences and distant metastasis. Obstructive symptoms of the involved nasal cavity and epistaxis are the most common initial symptoms. Other associated symptoms are rhinorrhoea, hyposmia, frontal headache, facial pain, proptosis, diplopia and epiphora [1, 5]. Our patient mainly presented with nasal discharge, rhinorrhoea and facial pain.

Primary diagnostic modality includes direct visualisation of the tumour using anterior rhinoscopy and nasolaryngoscopy, followed by doing a biopsy and radiographical imaging (contrast enhanced computed tomography (CECT) and MRI) to look at the extension of the disease and underlying distant metastasis. Immunohistochemistry is required to come to a definitive diagnosis as many other conditions may have a similar clinical presentation, like undifferentiated carcinoma involving the nasal cavity, lymphoma, rhabdomyosarcoma, angiosarcoma, neuroendocrine carcinoma, neuroblastoma and plasmacytoma, which were also considered in our patient [1]. Our patient was provisionally diagnosed with sinonasal malignant melanoma which was later confirmed by immunohistochemistry (IHC), showing positive HMB45 (other markers include S-100 protein, melan-A, microphthalmia-associated transcription factor, tyrosinase, vimentin and cytokeratin). Literature review done by Alves *et al* [1] stated that the relevant histological characteristics for prognosis in cases of malignant mucosal melanoma differ significantly from those of malignant skin melanomas. Features predictive of poor prognosis were vascular invasion, necrosis and a polymorphous population of tumour cells; while features like thickness of tumour, degree of invasion, ulceration, mitotic index and neural involvement had little effect on prognosis [1]. According to literature review done by Andrianakis *et al*. [6], nasal cavity primary tumours have a better prognosis than primary tumours originating from paranasal sinus possibly because of delayed diagnosis of paranasal sinus tumour due to its ‘hidden anatomic location’. Other significant prognostic factors are negative margin after resection which can be challenging due to complexity of the anatomy of sinonasal cavity and its proximity to vital structures, advanced stage, and interestingly, level of pigmentation.

Meta-analysis done by Gore and Zanation [2] and literature review done by Andrianakis *et al* [6] recommend that aggressive surgical resection of the tumour with confirmed free margins is the primary modality of treatment. With technological advances, endoscopic resections are comparable to external approaches. Adjuvant radiotherapy with a total radiation dose of 54 Gy or higher with standard fractionation schemes is mainly utilised for local control of disease with reduction in local tumour recurrence rate, but it does not have a significant impact on survival, and might be considered if margin status cannot be assessed. Retrospective analysis done by Caspers *et al* [7] evaluated the outcome of adjuvant radiotherapy in sinonasal mucosal melanoma and that done by Owens *et al* [5] did the same for mucosal melanoma of head and neck. Both analyses came to the same conclusion, which were consistent with literature, that adjuvant radiotherapy is associated with improved local control of disease but does not significantly improve survival. Chemotherapy and immunochemotherapy can be used for adjuvant treatment for distant disease and also neoadjuvant chemotherapy. Most of the trials studied by Gore and Zanation [2] used a single agent chemotherapy regimen, dacarbazine (DTIC) (used in our patient), temozolomide or vindesine [1, 5–7]. Anti-angiogenic therapies also play a role in the management of mucosal melanoma, including bevacizumab. Almost all neoplastic cells require angiogenesis for survival and metastasis which is commonly mediated by vascular endothelial growth factor (VEGF). Strong expression of VEGF is also seen in melanoma which allows it to be a highly angiogenic tumour. Hence, the use of anti-angiogenic therapy is a good and proven strategy with bevacizumab being extensively tested. Similarly, metronomic chemotherapy also works via anti-angiogenic mechanisms. OMCT utilises low dose systemic chemotherapy given for longer duration affecting the tumour microenvironment. Another important rationale behind the use of OMCT over bevacizumab is the financial factor with OMCT having lower financial consequences on patients. Also, the use of bevacizumab along with carboplatin and paclitaxel would render more toxic adverse events as compared to OMCT [11–13].

Based on these recommendations, our patient was initially managed by the surgical oncology team but was referred to medical oncology for palliative chemotherapy as the tumour was deemed unresectable. Dacarbazine was initially given, but the tumour progressed, and the patient was then given radiotherapy for local control of the disease, after which the patient was then shifted to OMCT, as intravenous chemotherapy was not feasible as the patient is elderly and immunotherapy was not feasible in view of financial constraints.

OMCT (consisting of Celecoxib, Cyclophosphamide and Tamoxifen) is a cheap and effective drug regimen used for palliative intent in patients who progress on systemic chemotherapy and are deemed non-curable. Metronomic cyclophosphamide has shown a good safety profile in elderly frail patients, as they have limited hospitalisation time and are able to stay at home for longer duration (our patient is on a 3 monthly follow-up). This regimen is well tolerated by the patients, with the main toxicity being lymphopenia (not yet seen in our patient) [8]. Oral metronomic cyclophosphamide has shown better progression-free survival (PFS) in patients with ECOG PS 1 (compared to PS 2) and those with locoregional recurrence (as compared to metastasis). These two variables were the only significant independent factors of PFS [9]. Due to the above said reasons, our patient was ultimately shifted to OMCT. A literature review done by Simsek *et al* [10] showed that the use of metronomic cyclophosphamide can be of benefit with slightly increased survival in those patients in which any other treatment modalities cannot be given. As stated earlier, the main mechanism of action is the anti-angiogenic effect of the OMCT, which plays a role in the pathogenesis of melanoma and also has a prognostic value [10].

## Conclusion

Sinonasal melanoma is a rare and aggressive tumour, with surgery being the primary modality of treatment. In patients whose tumours are deemed unresectable should be offered with systemic chemotherapy or immunotherapy, with radiotherapy used for local control of the disease. The fact that our patient showed a partial response while on OMCT is of significant value since OMCT is much cheaper and has a very small financial burden on patients, many of whom cannot afford other costly treatment modalities like immunotherapy and intravenous systemic chemotherapy.

## Funding

No funding was received for this study.

## Informed consent

Written informed consent was obtained from the patient for this study.

## Conflict of interest

The authors have no conflicts of interest to declare.
